# Emotional activation in video conferences equals that in in person meetings

**DOI:** 10.1177/20552076231183551

**Published:** 2023-06-20

**Authors:** Michelle Jerkku, Jonas Nordin, Niclas Kaiser

**Affiliations:** Department of Psychology, 8075 Umeå University, Umeå, Sweden

**Keywords:** Emotional activation, emotion processing, autobiographical recall, in person therapy, online therapy

## Abstract

**Objective:**

The purpose of this study was to increase our understanding of VCPs’ impact on the therapeutic factor *emotion processing* by investigating possible differences in emotional activation during autobiographical recall in VCPs and in person.

**Methods:**

We recruited 30 adult participants aged 21–53 (*M* = 26.50, *SD* = 6.68) with no current psychiatric diagnoses to join a controlled experiment. All participants completed two relaxation sessions and two autobiographical recall sessions. Each type of session was delivered once over a VCP and once in person. Emotional activation was measured by heart rate, skin conductance and self-assessment of affects during each session.

**Results:**

No significant differences in activation during autobiographical recall between VCP and in person.

**Conclusions:**

This result may indicate the viability of VCPs for work with emotion processing. We discuss the results in light of clients’ and therapists’ concerns about using VCPs in emotional work, with the caution that further practical implications should be considered.

## Introduction

The global COVID-19 pandemic led to greatly increased use of video conferencing platforms (VCPs) and substantial developments in remote healthcare. More and more people are receiving treatment online for physical and mental ailments. Swedish online healthcare provider Kry reported a 53% increase in clients from February to May in 2020,^1^ while a recent US survey showed a growing number of clients switching to, or beginning, therapy via VCPs.^2^ This surge in interest suggests the need for more studies of the new modality of therapy administration. The effects of specific components of VCP-delivered psychotherapy also require study.

Psychotherapy delivered by VCP software programmes such as Zoom, Microsoft Teams and Skype are often referred to as ‘online therapy’ and ‘cyber therapy’. We refer to these platforms collectively as VCPs and use *VCP-delivered therapy* to distinguish it from therapies delivered through text-based or non-synchronous platforms, as has previously described by Smith et al.^3^ We use the term *in person* for interactions where all parties are physically present in the same location.

Much of the existing literature on VCP-delivered therapy focuses on treatment outcomes. Research indicates that psychotherapy administered via VCP is effective for a range of treatment frameworks and diagnoses, with the most pronounced effects seen in cognitive behavioural therapy for anxiety disorders, depression and PTSD.^4^ Overall, treatment outcomes and dropout rates for such therapies are not inferior to in person psychotherapies.^5^ In a study by Thomas et al.,^6^ participants’ anecdotes indicate that VCP-delivered therapies might offer unique advantages, such as reduced costs and increased uptake of treatment, as clients do not need to be physically present at the clinic. Interventions may also become more ecologically valid as the therapy is delivered where the client's symptoms normally manifest.

However, VCP-delivered therapy may also have disadvantages. Clients tend to express a preference for in person therapy at the start of treatment, and some clients may find it difficult to adapt to the new medium.^6^ The lack of eye contact when using VCPs can affect nonverbal processes and create a sense of emotional distance.^7^ Technical difficulties such as delay or lag might also disrupt patients’ experiences of connection, engagement and being understood.^8^ The overall quality of treatment may also be affected by difficulties in interpreting nonverbal signals.^6^ The acceptance of VCPs is influenced by clients’ expectations and doubts, making it important for healthcare providers to ensure that these concerns are appropriately addressed.^9^

Despite these challenges, VCP-delivered therapy remains a promising treatment alternative. However, while much research exists on treatment outcomes, the literature is limited in regard to specific therapeutic factors. Most studies investigating therapeutic factors have focused on the therapeutic alliance. A recent meta-analysis by Norwood et al.^10^ on the therapeutic alliance in VCP-delivered therapy indicated its treatment outcomes were equal to in person treatment, but the strength of the alliance was slightly inferior. The researchers highlighted the need to continue investigating the alliance but also recommended further research on other aspects of treatment to better understand how the VCP modality influences therapy. Smith et al.,^3^ who share this view, recommend continued research on other therapeutic factors.

One such therapeutic factor is *emotion processing*, which several studies indicate may be important to final therapeutic outcomes.^11–14^ According to Lazarus and Fisher,^11^ the therapeutic outcome is affected by clients’ access to emotions both before and during therapy. Their study reports that emotion processing might be independent of the quality of the therapeutic alliance.

Emotion processing is central to many schools of therapy.^15^ For example, cognitive behavioural therapy stresses the interplay of emotions, thoughts and behaviours,^16^ while psychodynamic approaches emphasize the experience of specific emotions.^17^ Effective emotion processing is a significant aspect of treatment, since faulty emotion processing has been linked to maladaptive functioning and mental illness.^18^

Pascual-Leone and Greenberg designed a model that describes specific components of emotion processing throughout treatment.^12^ In this framework, emotion processing occurs in distinct sequences that give rise to gradual intrapersonal change during psychotherapy. Phase 1 starts with the experience of global distress or undifferentiated negative feelings. In phase 2, the client's emotional distress has been differentiated and is experienced as core maladaptive emotions such as shame or fear. During phase 3, the client reaches a sense of the self as deserving, which prompts them to address their unmet needs. According to this model, initial emotional activation is necessary to reach the later phases of emotion processing.^19^ Pascual-Leone and Greenberg's work would suggest that activating negative emotions in phases 1 and 2 is a prerequisite to achieving a satisfactory therapeutic outcome.

Another reason to focus on VCP-delivered therapy's effects on emotion processing is the role of emotions in making decisions. A 2015 meta-analysis by Lerner et al.^20^ concludes that emotions are pervasive, strong and predictable drivers of decision making. Pascual-Leone and Greenberg's model suggests that unprocessed and undifferentiated maladaptive emotions are prevalent in patients with mental illness. These emotions likely affect their interpretations and decision-making processes, which in turn affect their future actions during psychotherapy and their prospects of recovery.

An affect induction procedure (AIP), a form of intervention designed to elicit emotional activation, may be used in empirical studies of affect. In this paper, the terms *emotion* and *affect* are used interchangeably to refer to ‘a complex reaction pattern, involving experiential, behavioural, and physiological elements, by which an individual attempts to deal with a personally significant matter or event’.^21^ In a meta-analysis of 874 studies with 53,509 participants, AIPs were deemed generally effective,^22^ although the effects varied depending on the type of affect and the particular AIP.

One type of AIP is *autobiographical recall*, in which patients retell past events to re-experience the affects they felt at the time. Methods similar to autobiographical recall may also be used in psychotherapy, especially in psychodynamic therapies.^17^ Autobiographical recall is generally effective for inducing affects (general effect size, *g* = 1.36; negative affect, *g* = 1.34; and positive affect, *g* = 0.95). The effect size for inducing anger is large (effect size *g* = 2.50).^22^

Overall, clients and practitioners alike report subjectively different experiences with VCP-delivered and in person interactions.,^6,7^ Research indicates that the therapeutic alliance may remain similar across modalities, however, and it is conceivable that reported differences are attributable to shifts in specific therapeutic factors such as emotion processing. This makes it important to investigate how VCPs influence emotion processing during therapy.

Furthermore, controlled experiments on the efficacy of VCP-delivered AIPs are scarce, and we have not found any studies investigating emotion processing facilitated by another person via VCP. The purpose of this paper is to add to the growing knowledge of VCP-delivered psychotherapy by investigating emotion processing in interactions over VCPs. To this end, we explored possible differences in the initial stage of emotion processing, emotional activation, between VCP-delivered and in person.

### Research questions and hypotheses

Our research question was whether, during autobiographical recall of anger-inducing stories, differences in emotional activation exist depending on whether the recall is delivered via a VCP or in person. We had two hypotheses: (1) there are significant differences between emotional activation during VCP-narrated autobiographical recall and in person therapy, and (2) there are significant differences in emotional activation between the relaxation and autobiographical recall sessions conducted via VCP.

## Method

### Participants

We recruited participants through posters, posts on social media, emails to local organizations and schools and one researcher's personal network. Exclusion criteria were any current psychiatric diagnosis, age under 18 years, lack of vaccination against COVID-19 or self-reported anger issues. The sample included 30 participants (20 men and 10 women) aged 21 to 53 years (*M* = 26.50, *SD* = 6.68). Data on education, experience with VCPs and attitudes towards VCPs were collected (Table 1). As the study was conducted by psychology students completing their master's thesis on campus during the pandemic, most people who demonstrated interest were university students or faculty members. Of these, 25 had no prior relationship with the researcher conducting the autobiographical recalls; the remaining five were fellow master's students acquainted with both researchers and recruited to maximize the sample size. All tests were run with this subgroup included and excluded, and between-groups analysis showed their inclusion or exclusion had no significant effect on the results.

**Table 1.** Baseline characteristics of the participants.

	*N*	%
Gender		
Male	20	66.7
Female	10	33.3
Completed education		
High school	10	33.3
>2 years of university	20	66.7
Use of video conferencing platforms (VCPs) in the last 2 years		
A few times	3	10.0
At least once a month	2	6.7
At least once a week	9	30.0
Almost every day	16	53.3
First started using VCPs		
<1 year ago	3	10.0
1–2 years ago	15	50.0
>2–5 years ago	7	22.3
>5 years ago	5	16.7
Used VCPs for:		
School	28	93.3
Work	13	43.3
Talking to family and friends	20	66.7
To receive healthcare	2	6.7
Attitude to one-on-one interaction via VCPs		
Somewhat negative	5	16.7
Neutral	12	40.0
Somewhat positive	10	33.3
Very positive	3	10.0
Self-assessed ability to feel and identify own emotions		
Quite hard	4	13.3
Neither easy nor hard	4	13.3
Quite easy	20	66.7
Very easy	2	6.7
Self-assessed attitude towards getting angry		
Extremely uncomfortable	1	3.3
Very uncomfortable	4	13.3
Somewhat uncomfortable	8	26.7
A little uncomfortable	11	36.7
Not at all uncomfortable	6	20.0

### Instruments and materials

The primary outcome variables for emotional activation were physiological data (heart rate and skin conductance) and self-assessed emotion (positive affect, negative affect and basic affects).

### Physiological data

Heart rate and skin conductance are valid and reliable physiological measures of emotion, and both measures have been used in previous experiments that induce and measure anger.^23–25^ To record the participants’ heart rate and skin conductance, the Biopac system MP150 was used in combination with the wireless unit BioNomadix PPGED-R. Heart rate was measured by the PPGED-R oximeter placed on the participant's ring finger on the non-dominant hand. The oximeter uses light to measure changes in the concentration of blood, which is then used to calculate heart rate in beats per minute. Skin conductance was also measured using PPGED-R. Two disposable 2288-series 3M foam monitoring electrodes were placed on the participants’ index and middle fingers on the non-dominant hand and connected to the PPGED-R. Sigma electrode gel 100 was used on the electrodes. Skin conductance is measured by sending a weak current of 0.5 volts through the electrodes. The strength of the current varies as a result of changes in resistance due to fluctuations in the level of perspiration from the participant's fingers. Skin conductance is measured in microsiemens (*µ*S).

The physiological data were recorded using the software Acquisition 4.2 with a sample rate of 1000 Hz. Heart rate was computed from the oximeter input using the Acquisition 4.2's *find rate* function. This study employed a procedure similar to that of Marci et al.^25^ to decide relaxation and autobiographical-recall heart- rate values. The mean of the last 2 min was used for the relaxations and the mean of the last 4 min for the autobiographical recalls. The skin conductance data were filtered through a low-pass FIR filter fixed at 1 Hz using 4000 coefficients and a high-pass IIR filter fixed at 0.05 Hz. This is an established method of separating the phasic and tonic signals to create a separate channel containing spontaneous skin conductance responses.^26^ The filtered data were then subtracted from the original data to remove the spontaneous responses. The mean for the last 2 min of the relaxations and the last 4 min of the autobiographical recalls was then extracted from the new data channel and used in the statistical analyses. Physiological data were collected during the entire experiment.

### Self-assessed affect

The self-assessment consisted of an amended Swedish translation of the Positive and Negative Affect Schedule (PANAS). The PANAS has two scales, Positive Affect and Negative Affect and has good reliability and validity and is frequently employed in AIP studies.^27^ Participants rate how strongly they experience a particular emotion such as Interested, Guilty or Hostile on a Likert scale ranging from 1 (very slightly or not at all) to 5 (extremely). The scores are then totalled for positive and negative emotions, resulting in two final scores. Since there is no standardized Swedish translation of the PANAS, the researchers translated it themselves. To check for inconsistencies, two native English speakers fluent in Swedish then back-translated it. We made small amendments to the final translation after receiving their feedback.

The translated PANAS was supplemented with a separate section for Tomkins’ basic affects.^28^ As PANAS includes interest, fear and shame, the separate section contained anger, happiness, sadness, surprise, disgust and ‘dissmell’ (Tomkins's neologism for an infant's reaction to a noisome odour). Previous studies on emotion induction have similarly amended the PANAS.^29,30^ Our inclusion of Tomkins’ basic affects did not alter the original scales of the instrument and were treated separately from the supplementary basic affects during analysis. The supplementary items were rated using the same 1–5 Likert scale as PANAS. The main purpose of adding Tomkins’ basic affects was to measure whether anger was the primary emotion elicited by the autobiographical recall by comparing self-assessed anger to the other basic affects.

### Video conference platform

We elected to use Zoom as the VCP in this experiment because it is a popular service used in a wide range of private and professional contexts. Its basic features are typical of VCPs, and we deemed the software to have few idiosyncratic elements that could disturb the generalizability of its effects on emotional activation. We used standard settings for the experiment. Participants saw both themselves and the researcher on the screen.

## Procedure

**Figure 1.**
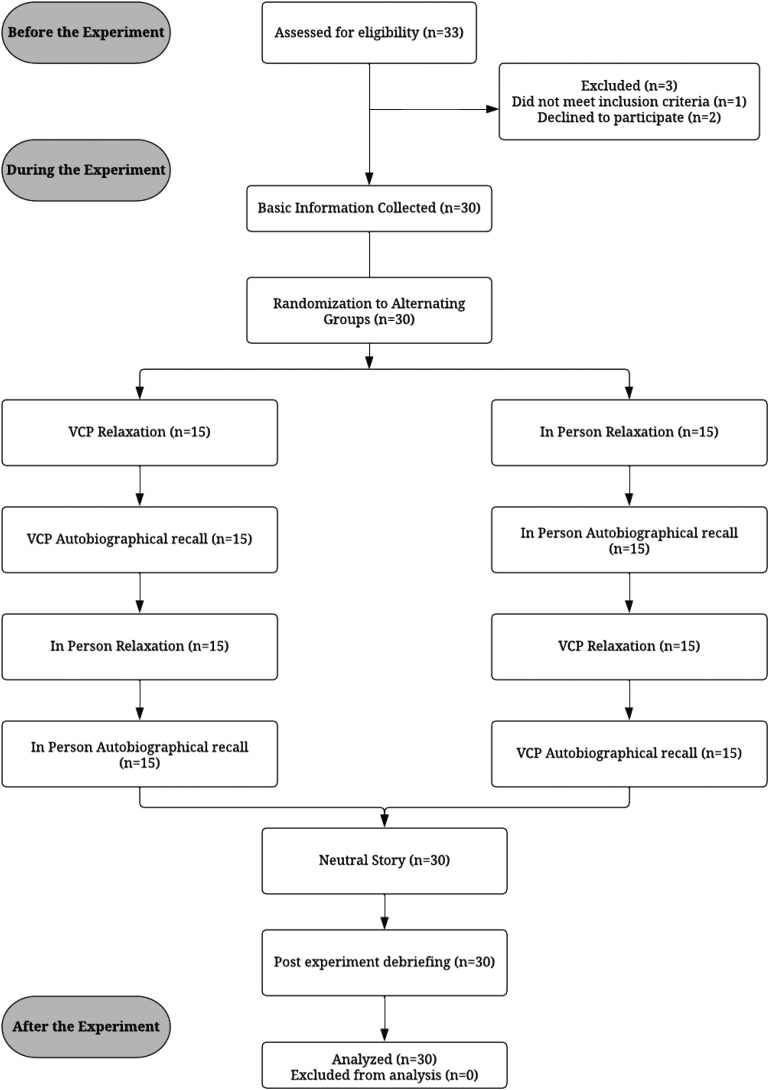
Flowchart of procedure and participant flow.

This experimental study used a repeated measures within-group design. For an overview of the full procedure, see Figure 1. Each participant completed five phases: relaxation 1 and autobiographical recall 1 using one modality, relaxation 2 and autobiographical recall 2 using the other modality and a brief neutral story in person at the end. Self-assessment using the amended PANAS followed each phase except the neutral story, which was not followed up with self-assessment because of time constraints. The study was counterbalanced to avoid any influence by order of conditions. This was done by randomly assigning participants to one of two groups. One group had their first two phases in person, and the other began with VCP sessions.

During their relaxations, participants were instructed to close their eyes and relax as they listened to 3 min of the song *Weightless* by Marconi Union. The music was played through speakers placed in the room in which they were seated. The song has been used in previous research to lower anxiety levels and help participants relax.^31^

During the autobiographical recall sessions, participants were instructed to recount an instance when they had felt anger and to try to re-experience the emotions they felt at the time. An experimenter asked questions from a standardized battery throughout their story. This was done to facilitate emotional activation, help participants talk about their story for the required 5 min and create an interaction similar to that of a patient and therapist during therapy. The battery was from a previous experiment by Burns et al.,^32^ and it included the following questions:

1. ‘How did that make you feel?’

2. ‘Can you tell me more?’

3. ‘What happened then?’

4. ‘What did that make you think?’

5. ‘What about the event made you angry?’

6. ‘What did the anger make you want to do?’

7. ‘How does it feel in your body?’

The researcher also responded with reflections such as ‘So that made you angry’, mirroring participants’ statements. Anger activation by autobiographical recall has been used in multiple studies.^25,33,34^

During the neutral story, to establish baseline physiological data, the researcher prompted participants to spend 60 s describing their usual route to the university. In this final session, the researcher sat in the room with each participant and asked no questions.

### Before the experiment

The day before the experiment, participants were advised to think of two instances that had made them angry and to make sure they were comfortable discussing them with the experimenter. They were encouraged to think of specific events of similar emotional intensity.

### During the experiment

**Figure 2.**
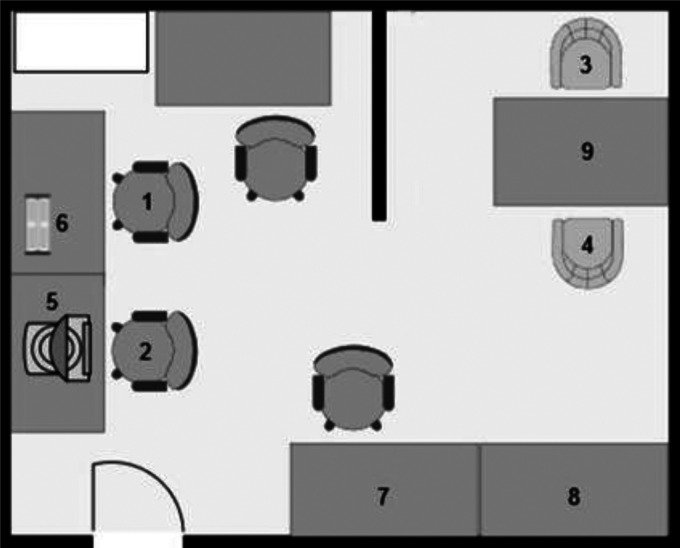
Setup of the room used during the experiment. *Note*: Standardized *z*-scores were calculated for each data point using the sample mean and sample standard deviation. *Note:* 1. Participant placement during the fitting of the Biopac equipment. 2. Researcher placement during fitting of the Biopac equipment. 3. Participant placement during both conditions. 4. Researcher placement during the in person condition and neutral story. 5. Laptop used for recording physiological data. 6. The Biopac 150 equipment. 7. Refreshments. 8. Speakers used to play relaxing music. 9. Laptop placement during VCP call.

***Pre-testing.*** The experiment took place in a lab at Umeå university during February 2022. For an overview of the room, see Figure 2. Participants came in alone and the entire procedure took approximately 1 hour. Researchers fitted participants with the equipment for measuring heart rate and skin conductance. Participants then moved to a part of the room designed to resemble a therapist's office. A screen blocked the view of the rest of the room, giving them no way of observing the computer used to record the physiological data. Participants sat in a chair by a table facing the researcher and completed a short questionnaire containing basic information. All participants wore the Biopac equipment for at least 5 min before the test as prescribed in an established protocol for measuring skin conductance.^26^ One researcher left the room once pre-testing was completed, leaving only the interviewing researcher to interact with participants during the experiment. Participants started with either the VCP or the in person condition depending on which group they had been assigned to.

***Testing: VCP.*** Participants were seated in front of a laptop computer with an active Zoom call. The researcher went to an adjacent room and joined the call. Internet connection on both computers was stable. Participants completed their 3-min relaxation and immediately filled out the amended PANAS. They then and recounted their autobiographical recall story for 5 min and again immediately filled out the amended PANAS.

***Testing: in person.*** The in person testing followed the same procedure as in the VCP condition except with no laptop as the researcher sat in the same room as the participants. Participants again completed their relaxation, filled out the amended PANAS, completed their autobiographical recall and filled out another amended PANAS self-assessment.

***Testing: neutral story.*** Participants were asked to relate a neutral story (their typical route to the university) for 60 s.

***After testing.*** Both researchers joined participants for a short debriefing. Participants were asked how they felt after the autobiographical recalls. They were also asked about any differences in their subjective experiences during their autobiographical recalls. Finally, they were given the chance to look at their physiological data and ask questions before the experiment concluded.

### Ethical considerations and participant anonymity

The Umeå University psychology programme exam board committee approved the study and assessed it as exempt from review by the national ethical committee.

All participants received oral and written information about the experiment and had an opportunity to ask questions. Prior to testing, participants signed consent forms and were informed that they were free to revoke their consent at any time. To ensure that the participants remained anonymous, all forms were coded using a random sequence of numbers. The consent forms, the basic information forms and the self-assessment forms were then stored separately in secure locations. The physiological data were kept on a USB stick in a locked safe. No identifying information tying the data to the participants was kept. Once the data from the forms had been analysed, all forms were shredded.

Since recollecting past events might be a painful experience in some circumstances, we took several steps to reduce the risk for the participants. First, the methods used were based on a tested and previously used affect-induction protocol. AIPs have been shown to have no long-term negative impact on participants’ moods. They merely cause a temporary increase in negative emotions, resulting in a somewhat lowered, though overall positive, mood. This tendency towards positivity that shields participants from a negative mood post procedure is referred to as the positivity offset.^22^ Second, we sought to mitigate the risk of adverse effects by excluding participants with psychiatric diagnoses and problems with anger, although there is no indication that people with psychiatric diagnoses respond more poorly than others to AIPs.^22^ Third, we repeatedly instructed participants to use events where they experienced *rejecting anger* (anger directed at someone or something other than themselves) because rejecting anger is a defence mechanism that protects the self from the painful experience of core maladaptive emotions such as shame and fear.^17^ Last, all participants were encouraged to talk about their experience during a short post-experiment debriefing and were offered additional support if needed. No participants reported feeling distressed at the end of the experiment.

### Data diagnostics and analytical strategies

All statistical analyses were conducted using SPSS Statistics 28 software. An alpha level of .05 was used for all statistical tests. The physiological data and positive and negative affect scales of PANAS were checked for normality, using Shapiro–Wilk, and for kurtosis and skewness. We also visually inspected the data. For kurtosis and skewness, normality was assumed if the *z*-converted scores were in the range of −2 to 2, in line with the recommendations of Hae-Young Kim.^35^ The data were analysed for outliers using SPSS's *explore* function. To compensate for outliers, analyses using variables with outliers were replicated with the outliers excluded.

We compared the physiological data and positive and negative affect scales during the four sessions (VCP relaxation, VCP autobiographical recall, in person relaxation and in person autobiographical recall) to detect differences. All normally distributed variables were compared using one-way within-group repeated ANOVA. The physiological data and positive and negative affect scales were used as dependent variables, and the four sessions were used as the within-participant factors.

When an ANOVA failed Mauchly's test of sphericity, estimates of sphericity were made using Field's recommendations.^36^ Greenhouse–Geisser's estimate of sphericity was used when *ε* < 0.75 and Huynh–Feldt's estimate when *ε* > 0.75.

Fisher's least significant difference was used for post hoc comparisons of the ANOVAs. This decision was based on recommendations from Armstrong,^37^ who argues that it is unnecessary to use Bonferroni's correction in small samples with relatively few tests. Using Bonferroni's correction decreases the risk of type I errors but increases the risk of type II errors. Type I errors are highly unlikely in our study given its small sample and relatively weak power, while type II errors are much more likely for the same reasons. All post hoc comparisons using Fisher's least significant difference were replicated using Bonferroni's correction. This resulted in a difference between skin conductance in the in person relaxation and autobiographical recall session, but no differences in the results.

Subtracting each participant's measurements during autobiographical recall from their measurements during relaxation resulted in a difference for each modality. We calculated the difference between VCP autobiographical recall and VCP relaxation to create a new variable: the VCP difference. The difference between the in person autobiographical recall and relaxation sessions was calculated to create a second new variable: the in person difference. These new variables were calculated for each participant for all physiological data and for the positive and negative affect scales. This means there were two new variables per participant for heart rate, skin conductance and self-assessed emotions based on their VCP and in person differences. Paired *t*-tests were then conducted on the new variables. This was done to investigate whether there were any significant individual differences between the overall VCP and in person differences.

The basic affects were treated as ordinal variables and analysed using Friedman's test to determine whether there were differences between the four relaxation and autobiographical recall sessions and to determine the effect size of those differences. Post hoc comparisons were made using individual Wilcoxon signed-rank tests. We compared the emotions with the highest and second highest effect sizes (the two basic affects with the greatest increase) using a Wilcoxon signed-rank test to determine whether one primary basic affect had been induced. The individual differences in basic affects were then made into new variables and compared in the same manner as the physiological data and the positive and negative affect scales. A Wilcoxon signed-rank test was then used to screen for significant differences. Post hoc power analysis was conducted using G*Power 3.1.9.7 to compute the achieved power in the repeated measures. ANOVA and paired samples *t*-tests were used for the physiological data and positive and negative affect scales. Results of the power analysis are presented in Table 2.

**Table 2.** Power analysis of heart rate, skin conductance and affect.

Outcome	ANOVA	Paired *t*-test	*Power*	*d*
	*Power*	*η* ^2^		
Heart rate	1	0.49	0.05	−0.01
Skin conductance	0.57	0.19	0.11	0.01
Positive affect	0.11	0.03	0.06	−0.06
Negative affect	0.99	0.46	0.09	0.11

*Note. n = 30.* Power calculated using G*Power. Effect size calculated using SPSS. The tests used did not allow the researchers to perform a power analysis for the basic affects.

## Results

All 30 participants completed the entire experiment. No data used in the main analysis were lost. Heart rate, skin conductance, positive affect and negative affect were deemed to be normally distributed, and tests were chosen accordingly. The variables anger, happiness, sadness, shame, disgust, dissmell, surprise, fear and interest (collectively referred to as basic affects) were treated as ordinal variables throughout the experiment, and tests were chosen accordingly. Two significant outliers were identified in the negative affect variable. All analyses using that variable were replicated with the outliers excluded, with no change in the significance of the results.

### Comparing relaxation to autobiographical recall

**Figure 3.**
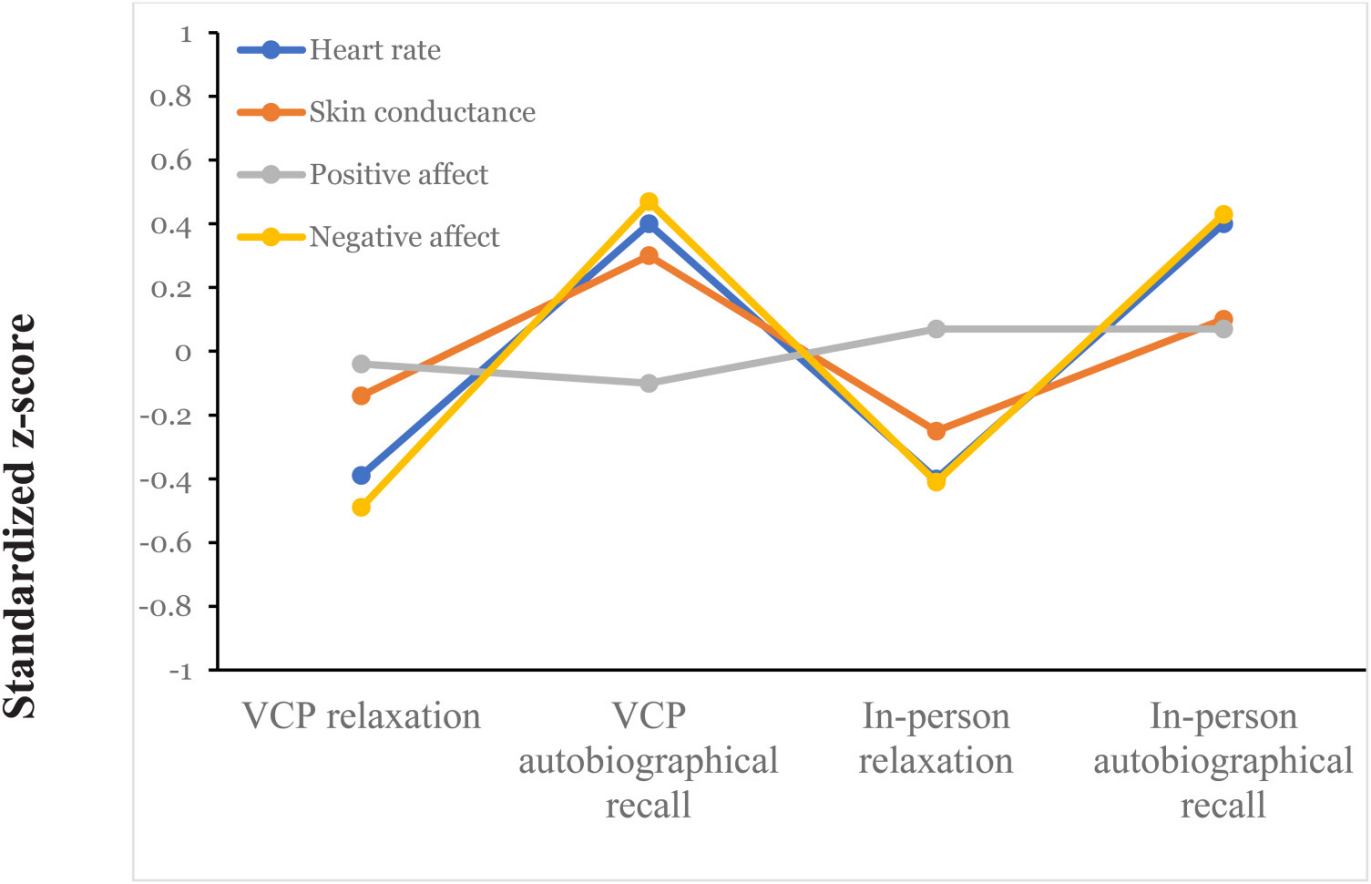
Standardized *z*-scores for the physiological data and affect across conditions.

**Figure 4.**
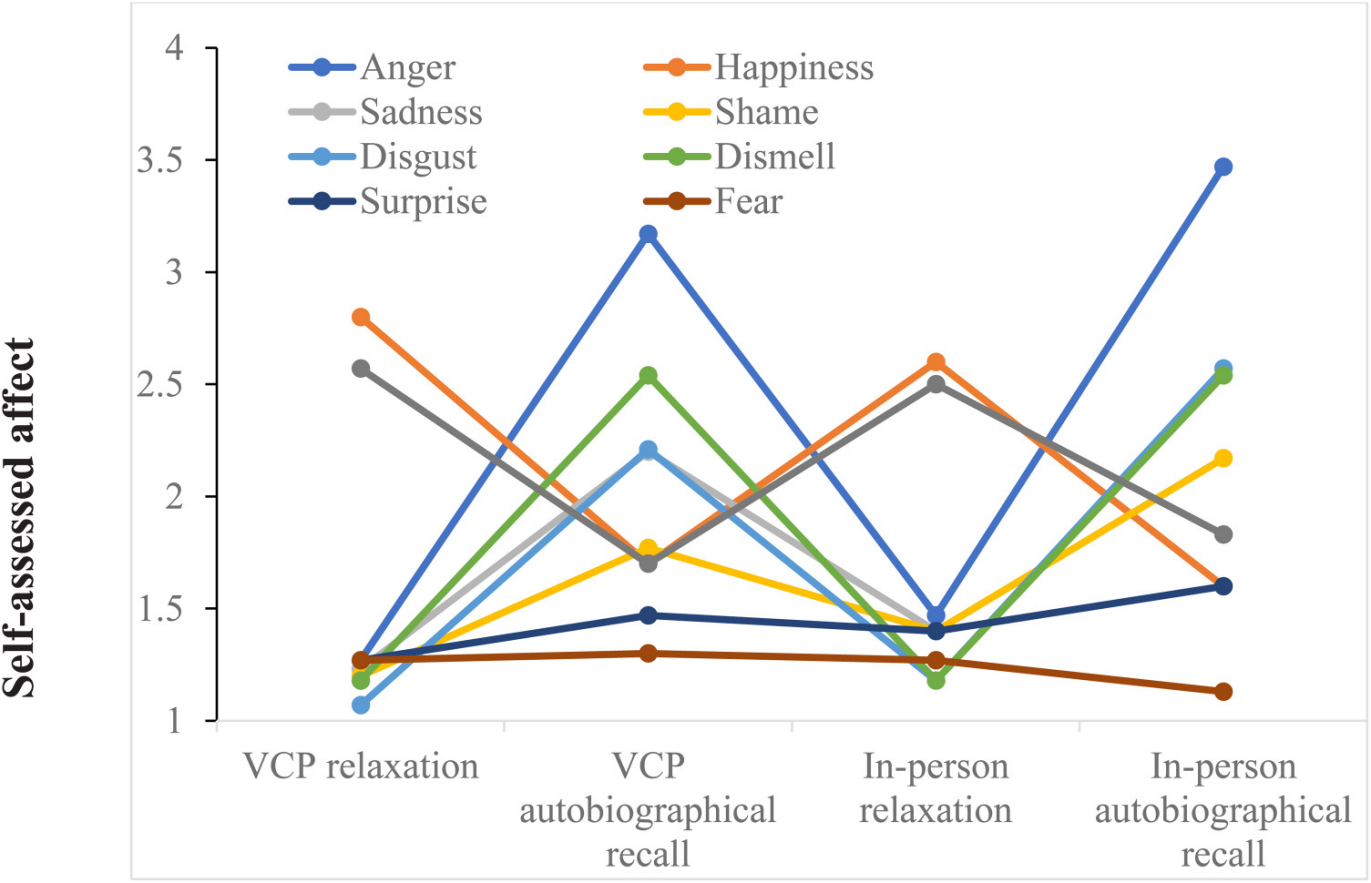
Tomkins’ basic affects across the conditions. *Note*: Self-assessment of basic affects across the four points of measurements: VCP relaxation, VCP autobiographical recall, in person relaxation and in person autobiographical recall. Self-assessment was done using a 5-point Likert scale ranging from 1 (very slightly or not at all) to 5 (extremely). VCP, video conferencing platform.

The physiological data and positive and negative affect scales were compared between relaxation and autobiographical recall using one-way repeated measures ANOVAs (Table 3) and visualized in Figures 3 and 4.

**Table 3.** Heart rate, skin conductance and affect across conditions.

	Phase								
	VCP relaxation		VCP autobiographical recall		in person relaxation		in person autobiographical recall		
Outcome	*M*	*SD*	*M*	*SD*	*M*	*SD*	*M*	*SD*	Statistics
HR (bpm)	72.04	11.64	83.32	14.19	71.89	11.74	83.27	14.72	*F*(3, 87) = 27.33, *p* < 0.001**
SC (*µ*S)	23.26	8.56	27.01	8.20	22.36	8.12	25.31	8.58	*F*(6.92^a^) = 0.19, *p* < 0.001**
Positive affect	21.60	6.34	21.20	7.59	22.40	6.79	22.40	6.48	*F*(0.79) = 0.03, *p = *0.505
Negative affect	14.33	4.17	19.17	4.43	14.70	5.52	19.00	4.01	*F*(24.18^b^) = 0.46, *p *< 0.001**

*Note.* SC and negative affect both increased significantly from relaxation to autobiographical recall measurements. Positive affect did not change.

HR, heart rate; SC, skin conductance; VCP, video conferencing platform.

**p < 0.001

^a^ Degrees of freedom corrected using Greenhouse–Geisser's estimate of sphericity (*ε* = 0.67).

^b^ Degrees of freedom corrected using Greenhouse–Geisser's estimate of sphericity (*ε* = 0.72).

***Heart rate.*** The one-way repeated measures ANOVA comparing the participants’ heart rates showed significant differences between the measurements (*F*(3, 87) = 27.33, *p < *0.001). Post hoc comparison using Fisher's least significant difference revealed significant differences between the VCP relaxation (*M* = 72.04, *SD* = 11.64) and the VCP autobiographical recall (*M* = 83.32, *SD* = 14.19, *p *< 0.001) and between the in person relaxation (*M* = 71.89, *SD* = 11.74) and the in person autobiographical recall (*M* = 83.27, *SD* = 14.72, *p *< 0.001). No significant differences were found between the VCP autobiographical recall and the in person autobiographical recall or between the VCP relaxation and the in person relaxation.

***Skin conductance.*** A one-way repeated measures ANOVA was performed to compare skin conductance between measurements for the participants. As Mauchly's test indicated that the assumption of sphericity had been violated (*χ*^2^(5) = 24.6, *p < *0.001), the degrees of freedom were corrected using Greenhouse–Geisser estimates of sphericity (*ε* = 0.67). The results showed a significant difference between the measurements (*F*(2.006, 58.160) = 6.92, *p = *0.002). Post hoc analysis using Fisher's least significant difference revealed a significant difference between the VCP relaxation (*M* = 23.26, *SD* = 8.56) and the VCP autobiographical recall (*M* = 27.01, *SD* = 8.20, *p *< 0.001) and between the in person relaxation (*M* = 22.36, *SD* = 8.12) and the in person autobiographical recall (*M* = 25.31, *SD* = 8.58, *p = *0.018). There were no significant differences between the in person autobiographical recall and the VCP autobiographical recall or between the in person relaxation and the VCP relaxation.

***Positive affect*.** The one-way repeated measures ANOVA indicated no significant differences in self-assessed positive affect between the four measurements (*F*(3, 87) = 0.79, *p = *0.505).

***Negative affect***. A one-way repeated measures ANOVA was performed to compare self-assessed negative affect between the measurements for the participants. As Mauchly's test indicated that the assumption of sphericity had been violated (*χ*^2^(5) = 16.8, *p = *0*.*005), the degrees of freedom were corrected using Greenhouse–Geisser estimates of sphericity (*ε* = 0.72). The results showed a significant difference between the measurements (*F*(2.167, 62.857) = 24.18, *p < *0.001). Post hoc analysis using Fisher's least significant difference revealed a significant difference between the VCP relaxation (*M* = 14.33, *SD* = 4.17) and the VCP autobiographical recall (*M* = 19.17, *SD* = 4.43, *p *< 0.001) and between the in person relaxation (*M* = 14.70, *SD* = 5.52) and the in person autobiographical recall (*M* = 19.00, *SD* = 4.01, *p < *0.001). There were no significant differences between the in person autobiographical recall and the VCP autobiographical recall or between the in person relaxation and the VCP relaxation.

***Basic affects.*** All basic affects were compared for differences between the measurements using Friedman's Tests (Table 4). The largest effect size was observed for anger (*w* = 0.81). Friedman's test showed significant differences in self-assessed anger between the measurements (*χ*^2^(3) = 72.43, *p* < 0.001). Post hoc analysis using Wilcoxon signed-rank tests indicated significant differences between the VCP relaxation (*Mdn* = 1, *IQR* = 0) and the VCP autobiographical recall (*Mdn* = 3, *IQR* = 2, *T* = 351, *z* = −4.52, *p *< 0.001) and between the in person relaxation (*Mdn* = 1, *IQR* = 1) and the in person autobiographical recall (*Mdn* = 4, *IQR* = 1, *T* = 435, *z* = −4.77, *p *< 0.001)*.* The difference between the in person autobiographical recall and the VCP autobiographical recall was not significant (*T* = 122, *z* = −1.72, *p = *0.088), nor was the difference between the in person relaxation and the VCP relaxation.

**Table 4.** Basic affects across conditions.

	Phase								
	VCP relaxation		VCP recall		in person relaxation		in person autobiographical recall		
Affect	*Mdn*	*IQR*	*Mdn*	*IQR*	*Mdn*	*IQR*	*Mdn*	*IQR*	Statistics
Anger	1	0	3	2	1	1	4	1	*χ*^2^(3) = 72.43, *p* < 0.001** *w *= 0.81
Happiness	3	1	2	1	3	2	2	1	*χ*^2^(3) = 52.75, *p* < 0.001** *w *= 0.59
Sadness	1	1	2	1	1	1	2	2	*χ*^2^(3) = 33.00, *p* < 0.001** *w *= 0.37
Shame	1	0	1.5	1	1	0	1	1	*χ*^2^(3) = 13.09, *p* < 0.004* *w *= 0.15
Disgust	1	0	2	2	1	0	3	1	*χ*^2^(3) = 54.11, *p* < 0.001** *w *= 0.64
Dissmell	1	0	3	1	1	0	2	2	*χ*^2^(3) = 52.37, *p* < 0.001** *w *= 0.62
Surprise	1	0	1	1	1	1	1	1	*χ*^2^(3) = 4.41 *p* < 0.221 *w *= 0.05
Fear	1	1	1	0	1	0	1	0	*χ*^2^(3) = 2.45, *p* < 0.485 *w *= 0.03
Interest	2	1	1.5	1	3	1	2	2	*χ*^2^(3) = 29.14, *p* < 0.001** *w *= 0.32

*Note.* Anger, sadness, shame, disgust and dissmell all increased significantly from baseline measurements to interview measurements. Happiness and interest decreased significantly from baseline to interview measurements.

*p < 0.05*.*

**p < 0.001.

To determine whether anger was the main basic affect induced by the autobiographical recalls, self-assessed anger was compared to self-assessed disgust, which had the second highest effect size of the basic affects (*w = *0.64). A Wilcoxon signed-rank test showed a significant difference between self-assessed anger (*Mdn* = 3, *IQR* = 2) and self-assessed disgust (*Mdn *= 2, *IQR *= 2) during the VCP autobiographical recall (*T* = 203.5, *z = *−3.8, *p* < 0.001) and between self-assessed anger (*Mdn* = 4, *IQR* = 1) and self-assessed disgust (*Mdn *= 3, *IQR *= 1) during the in person autobiographical recall (*T* = 182.5, *z = *−3.7, *p* < 0.001).

### Comparing the VCP and in person autobiographical recalls

Individual differences in physiological data and affect scales were compared using paired *t*-tests and Wilcoxon signed-rank tests (Table 5).

**Table 5.** Mean individual differences in heart rate, skin conductance, affect and anger.

	Means of individual differences between				
	VCP autobiographical recall and VCP relaxation		In person autobiographical recall and in person relaxation		
Outcome	*M*	*SD*	*M*	*SD*	Statistics
HR (bpm)	11.28	8.56	11.38	9.93	*t*(29) = −0.06, *p = *0.955, *d* = −0.01
SC (*µ*S)	3.75	3.63	2.96	6.44	*t*(29) = −0.71, *p = *0.483, *d* = 0.01
Positive affect	−0.40	5.02	0	5.27	*t*(29) = −0.33, *p = *0.744, *d* = −0.06
Negative affect	4.83	3.97	4.30	5.03	*t*(29) = −0.63, *p = *0.536, *d* = 0.11
Anger^a^	2	2	2	2	*t*(29) = −0.52^b^, *p = *0.603, *d* = 0.09

HR, heart rate; SC, skin conductance.

^a^ Median and interquartile range are reported instead of mean and standard deviation for anger.

^b^ Critical *z*-value from the Wilcoxon signed-rank test.

***Heart rate.*** The paired *t*-test comparing individual differences in heart rate between the VCP relaxation and the VCP autobiographical recall (*M* = 11.28, *SD* = 8.56) and the in person relaxation and the in person autobiographical recall (*M* = 11.38, *SD* = 9.93) showed no significant differences, (*t*(29) = −0.06, *p = *0.955).

***Skin conductance**.* The paired *t*-test comparing individual differences in skin conductance between the VCP relaxation and the VCP autobiographical recall (*M* = 3.75, *SD* = 3.63) and the in person relaxation and the in person autobiographical recall (*M* = 2.96, *SD* = 6.44) showed no significant differences (*t*(29) = −0.71, *p = *0.483).

***Positive affect*.** The paired *t*-test comparing individual differences in self-assessed negative affect between the VCP relaxation and the VCP autobiographical recall (*M* = 4.83, *SD* = 3.97) and the in person relaxation and the in person autobiographical recall (*M* = 4.30, *SD* = 5.03) showed no significant differences (*t*(29) = 0.63, *p = *0.536).

***Basic affects.*** The Wilcoxon signed-rank test comparing individual differences in anger between the VCP relaxation and the VCP autobiographical recall (*Mdn* = 2, *IQR* = 2) and the in person relaxation and the in person autobiographical recall (*Mdn* = 2, *IQR* = 2) indicated no significant difference (*T* = 107.5, *z* = −3.72, *p* = 0.603).

### Comparing the autobiographical recalls to the neutral story

To investigate whether the physiological activation during the autobiographical recalls was mostly attributable to normal activation when talking, the neutral story was compared with the VCP autobiographical recall and the in person autobiographical recall using the means of heart rate and skin conductance during the first minute of each measurement.

We compared heart rates during autobiographical recall and neutral story using a one-way repeated measures ANOVA. As Mauchly's test showed that the assumption of sphericity had been violated (*χ*^2^(2) = 6.79, *p = *0.034), the degrees of freedom were corrected using Huynh–Feldt's estimates of sphericity (*ε* = 0.86) The results indicated a significant difference between the measurements (*F*(1.72, 46.33) = 12.63, *p *< 0.001). Post hoc analysis using Fisher's least significant difference revealed a significant difference in heart rate between the neutral story (*M* = 80.65, *SD* = 13.03) and the in person autobiographical recall (*M* = 88.60, *SD* = 12.69, *p = *0.001) and between the neutral story and the VCP autobiographical recall (*M* = 89.45, *SD* = 14.13, *p < *0.001)*.*

Skin conductance during the autobiographical recalls and neutral story was compared using a one-way repeated measures ANOVA. It revealed no significant differences between the VCP autobiographical recall (*M* = 30.24, *SD* = 9.71), the in person autobiographical recall (*M* = 27.77, *SD* = 9.13) and the neutral story (*M* = 29.40, *SD* = 9.22).

## Discussion

The main purpose of this study was to add to the knowledge of emotion processing when using VCPs, which to our knowledge has not previously been studied. The viability of autobiographical anger recall via VCP was demonstrated. The results showed significant differences between VCP relaxation and VCP autobiographical recall for all physiological data and negative affects. In a comparison of all self-assessed basic affects during VCP autobiographical recall and VCP-delivered relaxation, anger had the highest mean, highest median and the greatest effect size in the autobiographical recall (*w *= 0.81). Our results indicate that autobiographical recall via VCP was indeed effective for anger as an AIP, even though the effect size was not quite as large as that mentioned in the literature.^22^

The pattern of the results of this study is consistent with previous literature indicating that therapy delivered via VCP might be comparable to in person therapy.^4,5^ Clients do experience VCP-delivered therapy differently to in person therapy,^3^ but it is difficult to detect precisely what factors contribute to this perceived difference. Our results indicate that emotional activation is not one of those factors.

Our results may contribute towards showing the viability of emotion processing via VCPs, as emotional activation is a prerequisite to processing core maladaptive emotions according to Pascual-Leone and Greenberg's sequential model of emotion processing.^12^ However, it is possible that other phases of emotion processing will be impacted by the medium.

Our participants, like those in previous studies, reported experiencing VCP-delivered sessions and in person sessions differently. Something unique seems to happen during in person interactions, but that something has been difficult to pin down in experiments. This subjective experience should be considered important, however, as it influences people's perceptions and expectations of using VCPs. The phenomenon may affect work in clinical settings, where therapists rely on multiple factors of interaction, such as body language and subtle changes in tone of voice. These therapists may struggle with a sense that something is different during VCP-delivered therapy if they do not understand the reasons for that experience.

While emotional activation does not seem to be affected by VCP delivery instead of in person, other phases integral to emotion processing might be. For example, the reconciliation of unmet needs and negative self-evaluations described in Pascual-Leone and Greenberg's sequential model might require the therapist to use other interactive factors to a greater extent than during the initial emotional activation. The therapist working via VCPs may therefore struggle to administer these portions of treatment as usual.

Differences between VCP and in person communications may or may not affect the interaction, but the awareness of an elusive difference may create effects of its own. That awareness might give rise to a sense of uneasiness in both therapist and client, possibly influencing the therapists’ ability to work with more complex aspects of emotion processing. If such effects do occur, they might prove challenging to capture in experiments. In a research setting, emphasis is typically placed on adhering to a standardized procedure, whereas therapy focuses on adequately addressing the client's experience. Following protocol does not place as high demands on accurately interpreting interpersonal signals, which means the researchers may be less likely to be affected during experiments. Thus, potential effects of uneasiness may not be detected.

Other factors related to an experimental setting might also mask possible differences between in person and VCP interactions. In our experiment, the participant and researcher got to know each other in person at the start of the experiment, creating a bond before the interaction began via VCP. This initial phase might have allowed the participants to feel connected to the researcher when they switched to using a VCP, possibly mitigating effects that would otherwise be present.^6^

It is also possible that emotional activation happens differently during unstructured conversations than during controlled interactions such as the autobiographical recall used in our experiment. However, even if there are differences, they might not necessarily affect therapy. The feeling that something is different has not been found to be related to any important therapeutic factor. This might explain why studies and meta-analyses consistently find no differences in therapeutic outcomes between in person and VCP-delivered therapies despite nearly ubiquitous reports from clients and therapists about experiencing them differently.

There are several potential limitations concerning validity and generalizability of this study. The first limitation involves the study design. We could not control for several confounding factors. For example, the participants told different stories during the autobiographical recall in each condition, which meant we could not control for the intensity of the activated emotions. Variations in physiological data may also be attributable to emotions or processes other than anger. Another confounding factor was the semi-structured nature of the interaction during the autobiographical recalls, which allowed for some variation between participants. We attempted to address this by having the same researcher perform all autobiographical recalls while closely adhering to scripted prompts. Further, we do not know whether the efficacy of this form of AIP depends on the quality of the interaction. Emotional activation may have been caused more by describing and remembering the anger-evoking encounter than by interacting with another person. That would mean that the emotional activation may not have been influenced by switching modalities. Finally, unblinded designs can allow potential biases in researchers and participants about the preferred outcome.

A second limitation concerns the relative homogeneity of the sample. Most participants were experienced VCP users in their 20s. They were mostly highly educated, had a positive attitude towards VCPs and had few issues with expressing anger. Finally, as this is a nonclinical sample, caution should be used in transferring these findings to a clinical setting.

A third limitation concerns the limited sample size (*N* = 30). It is possible that differences exist in emotional activation between VCPs and in person autobiographical recalls that our study failed to detect due to a lack of power. Similarly, there may be interaction effects that our study could not examine due to insufficient sample sizes. However, the differences that the study was able to detect should be robust, as they were found despite its limited power.

Despite these limitations, our results suggest practical implications. There is a persistent discrepancy between how clients and clinicians experience VCP-delivered therapy and its actual outcomes.^3^ We hope that studies such as ours may help assuage some concerns about possible adverse effects and drawbacks of the medium.

It would be useful to extend the current findings by further examining emotion processing, for example, by studying the reconciliation of core needs and negative self-evaluations that Pascual-Leone and Greenberg's model posits following the identification of core maladaptive emotions.^12^ Other general and specific therapeutic factors and other emotions and AIPs could also be interesting to study. Larger sample sizes might detect differences and interaction effects that we could not, and conducting research involving multiple interviewers might control for varying interviewer styles and abilities. Ecological validity might also be improved by investigating clinical samples.

Although more research is needed to show the generalizability of these results, the present study provides initial support for the equivalence of emotional activation during VCP and in person therapies, which can be considered a step towards establishing the feasibility of online emotion processing.

**Acknowledgments:** We would like to thank everyone who participated in the experiment and provided us with useful data and interesting insights into their experiences with VCP therapy. This study would not have been possible without you.

**Contributorship:** MJ and JN collected and analysed the data with help and supervision from NK; they also wrote the first draft of the manuscript, which was further developed in consultation with all authors. All authors contributed to the study's conception and design and read and approved the final manuscript. NK is the article's guarantor.
